# Acanthosis Nigricans Manifesting as a Paraneoplastic Syndrome Associated With Cholangiocarcinoma

**DOI:** 10.7759/cureus.35853

**Published:** 2023-03-07

**Authors:** Mariana Costa, Ana Valente, Andreia Freire Coelho, Sara Meireles, Miguel Barbosa

**Affiliations:** 1 Medical Oncology, Centro Hospitalar Universitário de São João, Porto, PRT

**Keywords:** cutaneous manifestation, early diagnosis, paraneoplastic syndrome, cholangiocarcinoma, acanthosis nigricans

## Abstract

We present the case of a 64-year-old woman with type 2 diabetes who was diagnosed with early-stage intrahepatic cholangiocarcinoma and underwent partial hepatectomy followed by adjuvant chemotherapy. The patient simultaneously developed skin lesions compatible with acanthosis nigricans (AN). Thirty-seven months after completing chemotherapy, the patient had a recurrence of extensive skin and mucosal lesions compatible with AN. A thoracic-abdominal-pelvic (TAP) CT showed a relapse with hepatic hilar adenopathy. Currently, she is under evaluation to undergo radical treatment. Malignancy is a rare cause of AN and skin lesions can arise before, during, or after the diagnosis. As a paraneoplastic syndrome, it is usually related to gastric adenocarcinoma, with cholangiocarcinoma being a rare entity in this setting. Although an uncommon manifestation, the malignant etiology should be considered among other prevalent causes, such as metabolic disorders, and establishing an association can lead to an early diagnosis and initiation of curative treatment.

## Introduction

Acanthosis nigricans (AN) is a common skin condition characterized by velvety and hyperpigmented plaques on the skin at intertriginous sites such as the neck and axillae. Other cutaneous or mucosal sites are less frequent [1]. This disorder is considered a cutaneous manifestation of a systemic disease, usually associated with insulin resistance. It is often related to obesity or other endocrine and metabolic disorders, such as diabetes. Malignancy is a rare cause of AN. Paraneoplastic AN is also known as acanthosis nigricans maligna (ANM). In ANM, the cutaneous lesions can arise before, during, or after the tumor diagnosis [1].

Gastric adenocarcinoma is the most frequent tumor associated with ANM, but other tumor sites have also been described [2-3]. However, the association with cholangiocarcinoma is rare with only a very few cases reported in the literature so far [4-5]. Usually, these patients are older than 40 years and not obese, and this condition has no predilection for a particular sex or race. Other features associated with ANM are rapid onset and extensive skin involvement, lesions in atypical sites (mucous membranes, palms, or soles), unexplained weight loss, and other paraneoplastic findings such as the Leser-Trélat sign or tripe palm. ANM can be associated with localized or generalized pruritus and cutaneous and mucosal papillomatosis. There is no specific treatment approach for this condition, and the therapeutic management of the underlying malignancy usually leads to the resolution of the skin lesions [2,3].

Cholangiocarcinoma is a rare tumor with a particularly poor prognosis. Only 30% of the patients are diagnosed with resectable disease and, of these, 60% experience a post-surgical relapse. Thus, an early diagnosis is pivotal for initiating treatment with curative intent [6].

## Case presentation

**Figure 1:**
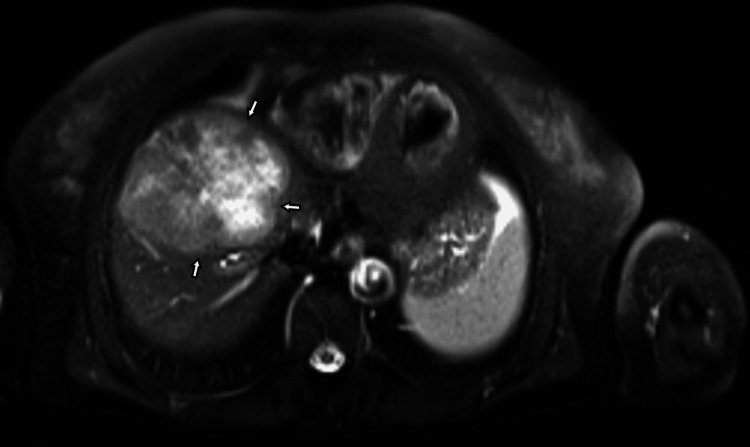
Abdominal and pelvic MRI showing a large hepatic nodule MRI: magnetic resonance imaging

**Figure 2:**
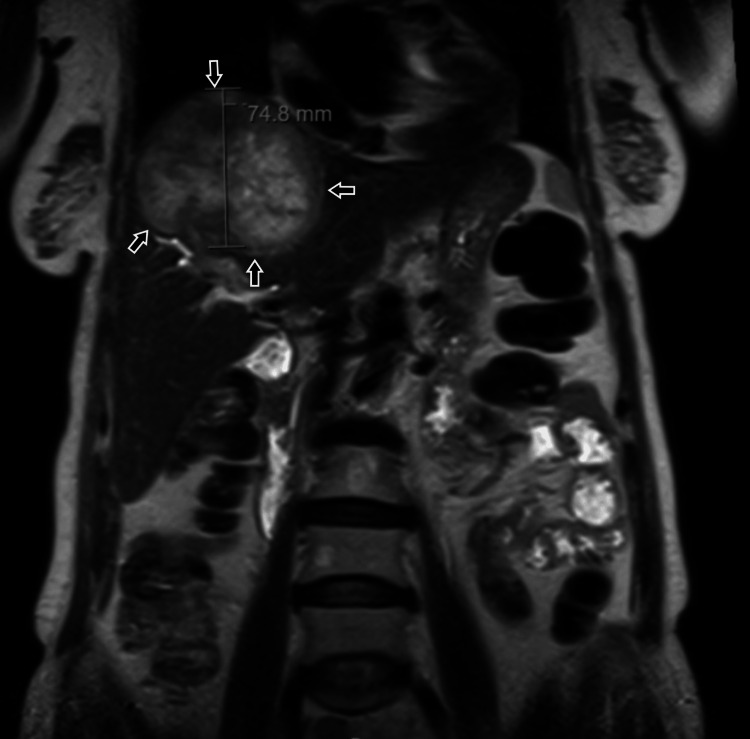
Abdominal and pelvic MRI with large hepatic nodule - coronal view MRI: magnetic resonance imaging

A 64-year-old-woman, a factory worker with a past medical history of metabolic syndrome (type 2 diabetes with poor metabolic control, hypertension, and dyslipidemia) and cerebrovascular disease, presented with complaints of darkened skin in the armpits and neck along with considerable weight loss (12% of body weight) in the past year. The laboratory workup revealed cholestasis. A thoracic-abdominal-pelvic (TAP) CT scan was performed, which revealed a large hepatic nodule (88 x 74 x 65 mm), without any further suspicious lesions. The abdominal and pelvic MRI confirmed the presence of this lesion (Figures 1, 2).

The liver biopsy was compatible with biliopancreatic carcinoma. Regarding the tumor markers, serum CA 19.9 was elevated (63 U/mL; normal range: <37 U/mL), with a normal carcinoembryonic antigen (CEA). The case was discussed at the Multidisciplinary Hepatobiliary and Pancreatic Tumours Board and surgery with curative intent was proposed. A partial hepatectomy was performed (December 2017) and the pathological report showed poorly differentiated intrahepatic cholangiocarcinoma (pT2aN0R0). After surgery, the serum CA 19.9 normalized and the skin lesions improved. During the cholangiocarcinoma diagnosis and treatment, the dark skin lesions were assumed to be AN secondary to poorly controlled type 2 diabetes.

**Figure 3:**
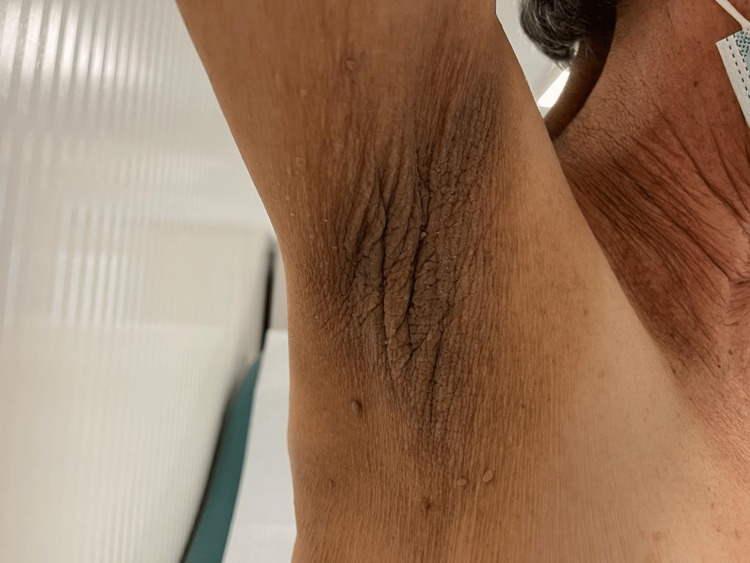
Acanthosis nigricans localized at axillae

**Figure 4:**
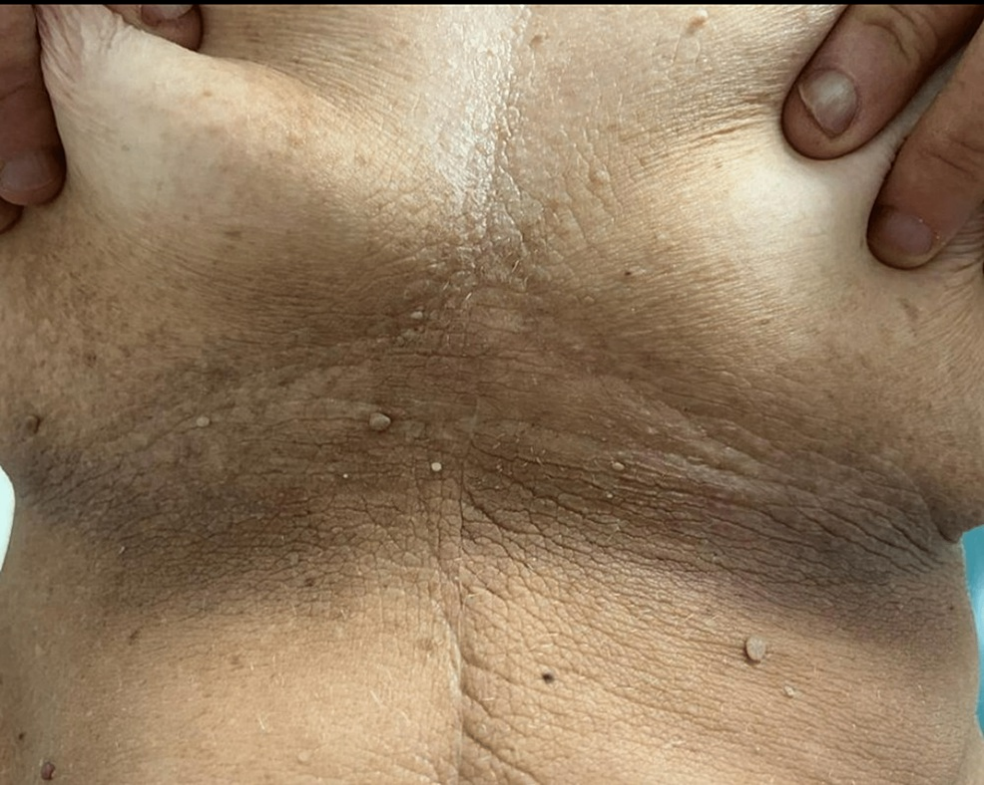
Acanthosis nigricans localized at the mammary sulcus

**Figure 5:**
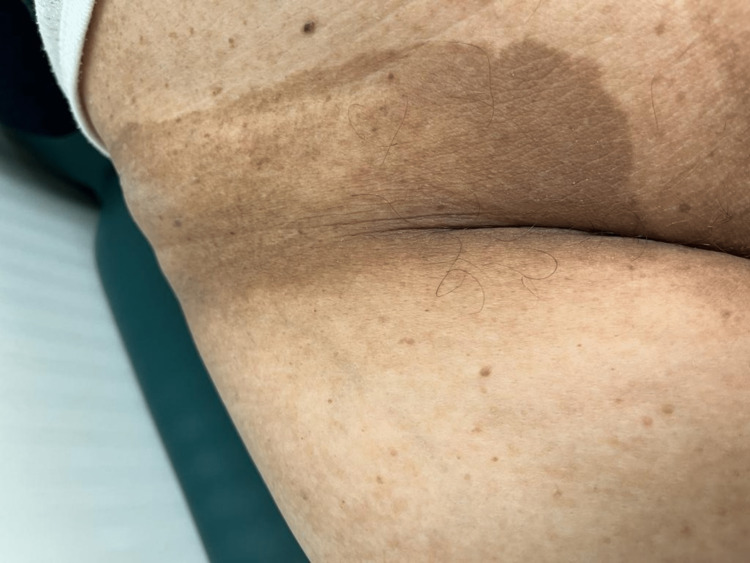
Acanthosis nigricans localized at the vulva

After adjuvant chemotherapy, the patient underwent regular follow-ups. In December 2022, she reported a rapid onset of dark skin lesions (like those presented at the time of the initial diagnosis) in the past month. Physical examination revealed extensive lesions of AN scattered throughout the body (localized mainly at axillae, mammary sulcus, and vulva; Figures 3, 4, 5, respectively). Serum CEA and CA 19.9 were normal. The TAP CT showed *de novo* bilateral pulmonary nodules (the largest one measuring 13 x 8 mm) and a large hepatic hilar adenopathy.

The patient was evaluated at a Dermatology appointment, where the lesions were described as brown plaques with epidermis hyperplasia and papillomatosis consistent with AN. Since the clinical features were highly suggestive, a cutaneous biopsy was deemed unnecessary. Besides, taking into account a direct temporal link to her cancer relapse, a malignant etiology was established.

**Figure 6:**
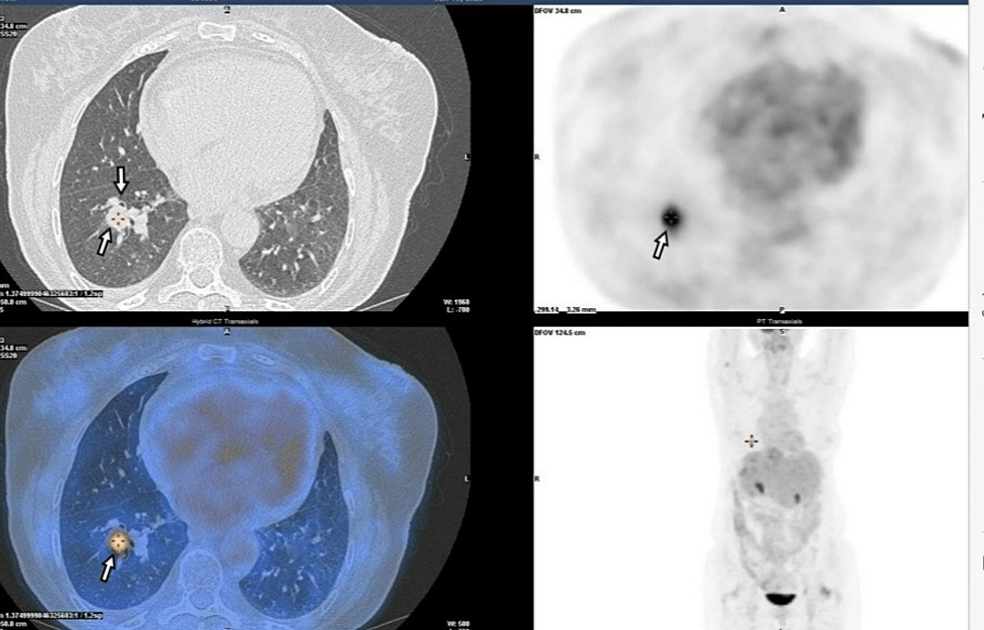
FDG-PET with mild uptake on the pulmonary nodule. The pulmonary biopsy was compatible with pulmonary anthracosis FDG-PET: fluorodeoxyglucose positron emission tomography

A pulmonary biopsy was performed, which revealed pulmonary anthracosis. A fluorine-18-fluorodeoxyglucose positron emission tomography (FDG-PET) was also done and showed a mild uptake on pulmonary nodules, in line with their inflammatory etiology (Figure 6). On the other hand, it also exhibited a high uptake at the large hepatic hilar adenopathy, validating a loco-regional relapse.

Currently, the patient is under evaluation by the Multidisciplinary Hepatobiliary and Pancreatic Tumours Board regarding a loco-regional curative treatment for the recurrence.

## Discussion

Cholangiocarcinoma is an aggressive tumor and its incidence is on the rise in Western countries. Typically, it is asymptomatic in the early stages and is often diagnosed as an advanced disease, thereby compromising the opportunity for a curative treatment [6]. AN is a common benign cutaneous disorder and it is easily diagnosed on physical examination. AN is also frequently associated with metabolic diseases, such as diabetes. When it presents as a paraneoplastic syndrome, ANM is normally related to gastric adenocarcinoma [1-3].

In our clinical case, the patient had AN manifestation as a paraneoplastic syndrome secondary to cholangiocarcinoma. AN's association with this tumor has been very rarely described and the patient's type 2 diabetes diagnosis was a confounding factor to the etiology of AN. However, an unexplained weight loss and the appearance of extensive lesions in our patient make the paraneoplastic syndrome hypothesis more likely [2-3]. The recognition of AN as paraneoplastic syndrome in our patient at the last recurrence led to prompt restaging and diagnosis, enabling the initiation of treatment with curative intent.

Our case sheds light on the role of AN as a paraneoplastic syndrome. It also underlines the impact that an expeditious acknowledgment of a paraneoplastic syndrome could have on the prognosis of a dismal tumor. Despite being rare, a malignant etiology should always be on the list of potential causes of AN. A diagnosis of ANM can lead to an early diagnosis of cancer, enabling the initiation of immediate treatment of an aggressive disease. Fortunately, in our patient's case, her cholangiocarcinoma recurrence will probably benefit from a curative approach.

## Conclusions

AN is a rare paraneoplastic syndrome and its association with cholangiocarcinoma has been scarcely reported. In our case, the patient's type 2 diabetes medical history was an initial confounding factor with regard to the etiology of AN. Despite being rare, a malignant etiology should always be part of the list of potential causes of AN. Prompt recognition of AN can lead to an earlier diagnosis and a higher chance of providing a curative treatment. This is crucial in the setting of cancers with a dismal prognosis such as cholangiocarcinoma.
